# The neuronal and synaptic representations of spatial release from masking in the rat auditory cortex

**DOI:** 10.3389/fnins.2025.1562183

**Published:** 2025-05-14

**Authors:** Guanhua Chen, Jiping Zhang

**Affiliations:** Key Laboratory of Brain Functional Genomics, Ministry of Education, NYU-ECNU Institute of Brain and Cognitive Science at NYU Shanghai, School of Life Sciences, East China Normal University, Shanghai, China

**Keywords:** spatial release from masking, detection threshold, auditory cortex, excitatory postsynaptic current, rats

## Abstract

In complex acoustic environments, both humans and animals are frequently exposed to sounds from multiple sources. The detection threshold for a target sound (or probe) can be elevated by interference sounds (masker) originating from various locations. This masking effect is reduced when the probe and masker are spatially separated compared to when they are colocalized, thereby improving the perception of the probe. This phenomenon is known as spatial release from masking. Currently, the neuronal and synaptic mechanisms underlying spatial release from masking in the auditory cortex are not fully understood. Here we employed single-unit recording and *in vivo* whole-cell patch-clamp recording techniques to examine how maskers from different spatial locations influence the detection thresholds of rat primary auditory cortex (A1) neurons in response to probe stimuli. At the cortical neuronal level, the masked detection thresholds of most A1 neurons in response to probes were significantly decreased when maskers were displaced from azimuths colocalized with the probe to other separated azimuths ipsilateral to the recording site. Similarly, at the cortical synaptic level, the masked detection thresholds of A1 neurons, as determined from the amplitude of evoked excitatory postsynaptic currents in response to probes presented at azimuth locations within the contralateral hemifield, were also decreased when maskers were shifted from azimuth locations in the contralteral hemifield to those in the ipsilateral hemifield. This study provides neuronal and synaptic evidences for spatial release from masking in the auditory cortex, advancing our understanding of the mechanisms involved in auditory signal processing in noisy environments.

## 1 Introduction

In natural acoustic environments, both humans and animals are frequently exposed to sounds from multiple sources. Sounds originating from the same or different directions may arrive at the two ears either simultaneously or sequentially. The auditory system possesses the remarkable capability to segregate target sounds (or probes) from interfering background noise, which is essential for effective perception of probes and successful communication in noisy environment. Previous research has shown that the detection threshold for a probe can be elevated by interference sounds (or maskers), known as masking (Soderquist et al., 1981; Alexander and Lutfi, 2004; Liang et al., 2014). When the probe and masker are spatially separated, this masking effect is attenuated compared to when they are colocalized, thereby improving the perception of the probe (Saberi et al., 1991; Freyman et al., 1999; Hawley et al., 1999). This phenomenon is referred to as spatial release from masking.

The degree of spatial release from masking can vary depending on factors such as the frequency and level of the sounds, the spatial configuration of the sound sources, and the listener's auditory capabilities and experience (Kidd et al., 1998; Middlebrooks, 2017). The effect of spatial release from masking has been observed in human in both children (Hess et al., 2018; Corbin et al., 2021) and adults (Kidd et al., 1998). Similar findings have also been reported in non-human primates (Rocchi et al., 2017) and other animals, such as guinea pig (Greene et al., 2018), ferrets (Hine et al., 1994), mice (Hine et al., 1994), bats (Warnecke et al., 2014), and frogs (Nityananda and Bee, 2012; Ward et al., 2013). In primates, the effect of spatial release from masking is more pronounced for azimuthal separations compared to elevation separations (Rocchi et al., 2017). In frogs, spatial release from masking improves the performance in sound pattern discrimination (Ward et al., 2013) and source identification task (Nityananda and Bee, 2012). Overall, the spatial release from masking enhances the perception of speech in humans and communication sounds in animals in complex acoustic environments.

Several previous studies in animal models have investigated the neural correlates of spatial release from masking within the ascending auditory pathway. In the frog inferior colliculus, a subset of neurons exhibited improvements in signal detection thresholds as the angular separation between probe and masker sources increased (Ratnam and Feng, 1998). This effect was more pronounced in the frog midbrain compared to auditory nerve fibers, indicating that central processing mechanisms contribute to spatial release from masking (Lin and Feng, 2001). Additionally, GABAergic interaction plays an important role in this phenomenon within the frog midbrain (Lin and Feng, 2003). In the cat inferior colliculus, while individual low-frequency neurons sensitive to interaural time differences did not consistently show improved masked thresholds with increasing separation between signal and masker, the population-level masked thresholds for these neurons did improve with signal and masker separation as a result of the variety of azimuth preference (Lane and Delgutte, 2005). At the level of auditory cortex, mutual information in the label line code of a subset of mouse cortical neurons increases as the spatial separation between the probe and masker enlarges (Nocon et al., 2023). In the songbird auditory cortex, the performance of encoding song identity at a given site was the best when the probe was presented contralaterally and the masker was presented ipsilaterally relative to the recording site (Maddox et al., 2012). Despite these findings, the cortical mechanisms underlying spatial release from masking remain only partially understood. Specifically, the synaptic representation of this phenomenon has yet to be elucidated. By employing single-unit recording and *in vivo* whole-cell recording techniques, the present study aims to systematically explore the neuronal and synaptic representations of spatial release from masking in the rat auditory cortex, thereby advancing our understanding of this phenomenon.

## 2 Materials and methods

### 2.1 Animals and surgery

Sprague-Dawley (SD) rats (8–10 weeks age) were used in this study. The rats were purchased from Shanghai Jie Si Jie Laboratory Animal Co., Ltd, and were reared in a room (room temperature, 20–24°C) with a 12 h light/dark cycle. The rats had free access to food and water. The experimental procedures were approved by the Institutional Animal Care and Use Committee (IACUC) of East China Normal University. All efforts were made to minimize the suffering of rats and the number of rats used.

Before surgery, the rats were anesthetized with urethane (intraperitoneal injection, 1.5 g/kg body weight). Subsequently, atropine sulfate (subcutaneous injection, 0.01 mg/kg body weight) was administered to the rats to reduce bronchial secretions. Additional doses of urethane were injected as needed to maintain anesthesia during the experiment. The rats' body temperature was maintained at 37°C using a feedback-controlled heating blanket. Following tracheal cannulation, the dorsal skull and a portion of the left temporal skull of the rats were surgically exposed. A 4-cm-long nail was securely affixed to the frontal dorsal skull by 502 super glue and dental cement for head fixation during physiological recording. A craniotomy was performed on the left temporal skull over the auditory cortex based on the stereotaxic coordinates specific to rat brain (Paxinos and Watson, 2007). Part of the dura on the cortex was removed to expose the left auditory cortex. Warm saline was applied onto the exposed brain to prevent drying. The rats were sacrificed at the end of electrophysiological recording by overdose injection of urethane.

### 2.2 Acoustical stimulation in electrophysiological studies

**Figure 1:**
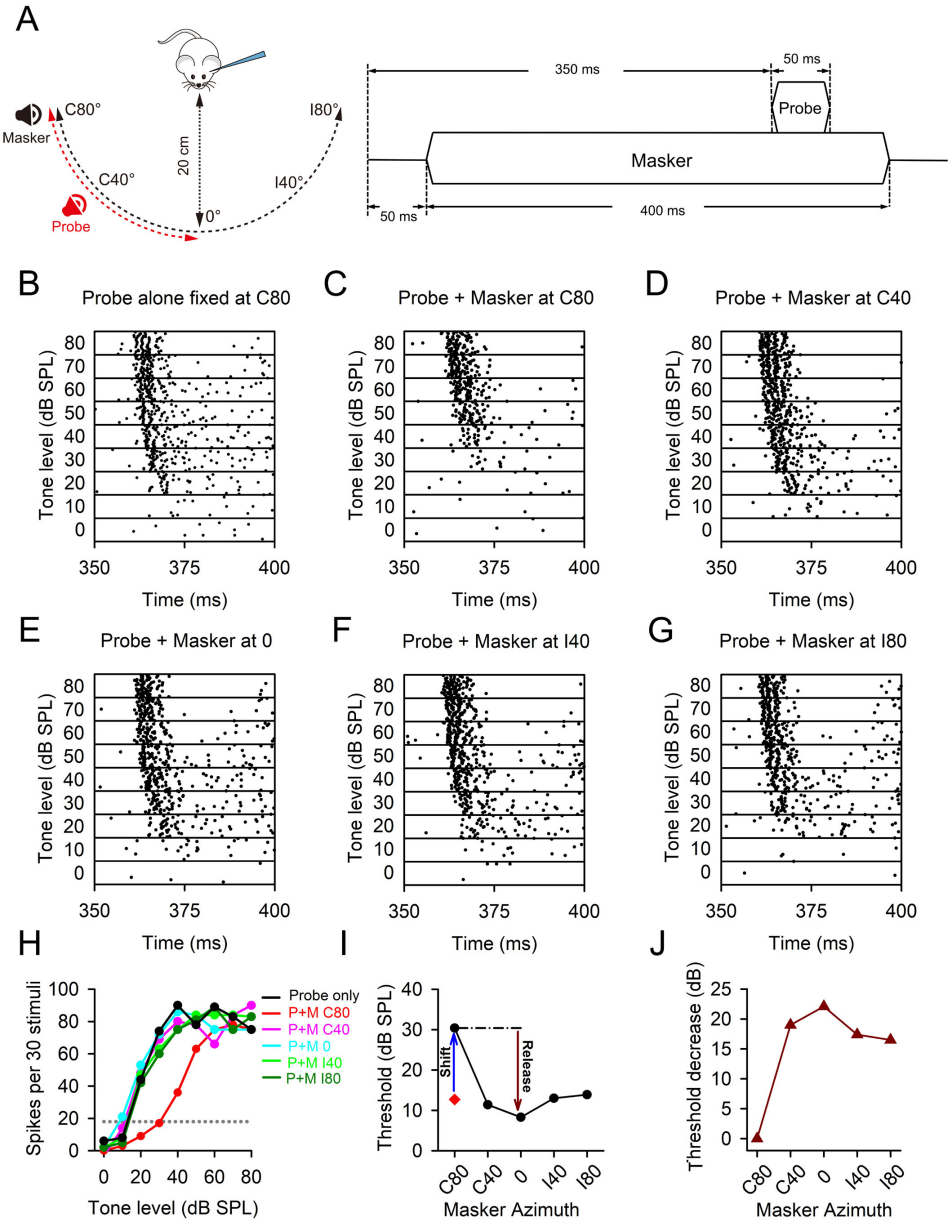
The data of a representative A1 neuron demonstrating spatial release from masking. **(A)** The schematic plot of the masker and probe configurations used in this study. The spatial azimuths of the masker were varied systematically from C80 to C40, 0, I40 and I80, while the spatial azimuths of the probe were varied from C80 to C40 and 0. In each trial, the masker (noise) starts 50 ms after the beginning of the trial, while the probe (pure tone) starts at 350 ms after the begining the trial. **(B–G)** Dot raster plots that illustrate the response of an A1 neuron to different stimulus conditions: probe-only condition **(B)**, and probe + masker conditions while varying the spatial azimuth of the masker. The spatial azimuths of the masker were contralateral 80° (C80, **C**), contralateral 40° (C40, **D**), frontal 0° (0, **E**), ipislateral 40° (I40, **F**) and ipislateral 80° (I80, **G**) respectively. **(H)** The rate-level functions of the A1 neuron determined under different stimulus conditions. Dotted line is the reference line showing 20% of the maximum response under probe-only condition. The detection threshold of the neuron at each condition was determined from the sound level at which the reference line intersects with the corresponding rate-level function. **(I)** The detection threshold, threshold shift, and the release of masking when the masker azimuth was varied. **(J)** The threshold decrease determined at different masker azimuths relative to the threshold measured when the azimuths of the masker and the probe were aligned.

Acoustical stimuli were presented in free-field using an auditory neurophysiology workstation (TDT3, USA), which consisted of a multifunction processor (RX6-A5), an electrostatic speaker driver (ED1), and two free-field electrostatic speakers (ES1). The outputs of the two ES1 speakers were calibrated from 4.0 to 44.0 kHz (sampling rate, 100 kHz) using a 1/4-inch condenser microphone (model 7016, ACO Pacific Inc.). The calibration data were stored in the computer for obtaining desired sound pressure level (dB SPL) within the calibrated frequency range. In this study, pure tone stimuli (50 ms duration, 5 ms rise time and 5 ms fall time) were used as probes. Broadband white noise bursts (4.0–44.0 kHz spectrum range, 400 ms duration, 5 ms rise time and 5 ms fall time) were used as maskers. The probes and maskers were independently delivered through two ES1 speakers that could be varied freely in azimuth positions. The azimuth positions for the speaker presenting the masker were located in contralateral hemifield at 80° (C80) and 40° (C40), frontal 0° (0), and ipsilateral hemifield at 40° (I40) and 80° (I80), relative to the recording site of neurons in the brain. Additionally, the azimuth positions for the speaker presenting the probe were set at C80, C40, and 0 (Figure 1A, left). To avoid potential collisions of the two ES1 speakers when they are moved to the position with identical horizontal and vertical coordinates, the elevation angle for the speaker presenting the probes was set to 0°, while the elevation angle for the speaker presenting the maskers was consistently set to 10° across all the tested conditions. For each stimulus trial, the masker (400 ms duration) was started from 50 ms after the beginning of each trial, and the probe (50 ms duration) was started from 350 ms after the beginning of each trial (Figure 1A, right). The probe was temporally overlapped with part of the masker. The inter-trial-interval was 1,200 ms.

### 2.3 Single-unit recording

The responses of rat A1 neurons to sound stimuli were recorded in a double-wall soundproof room. Glass electrodes (with an impedance of 1.0–2.0 MΩ, filled with 2 M NaCl) were used for single-unit extracellular recording to capture neuronal signals. The electrodes were precisely advanced perpendicular to the auditory cortex using a remote-controlled microdrive (SM-21, Narishige, Japan). The neuronal signals underwent amplification (1,000 × ) and band-pass filtering (0.3–3.0 kHz) through a preamplifier (DAM80, WPI, USA), followed by further signal processing via an adjustable gain preamp (GA8GA) and a medusa base station (RA16) for signal processing. Additionally, the amplified electrode signals were simultaneously monitored audiovisually through both a digital oscilloscope (TDS 2024, USA) and an audio speaker. The data of neuronal signals were stored in a computer for both online and offline analyses.

The localization of the rat primary auditory system (A1) was determined based on the stereotaxic coordinates (Paxinos and Watson, 2007), as well as physiological criteria such as a high-to-low gradient of characteristic frequency (CF) of neurons from anterior-to-posterior, short-latency responses to CF stimuli, and sharp frequency tuning (Zhang et al., 2001; Doron et al., 2002). Preliminary electrode penetrations were conducted in the auditory cortex of each rat to search for a tonotopic organization, characterized by a decrease in CFs along the anterior-posterior axis. The CF of an auditory cortex neuron was defined as the tonal stimulus frequency that evoked a response at its lowest response threshold (Minimum threshold, MT). The searching for the tonotopy in the rat auditory cortex was conducted by determining the CFs of cortical neurons through audio-visual observation. Once the A1 boundary was roughly delineated for each rat, subsequent single-unit extracellular recording was performed within the A1 to determine individual neuron's CF using a frequency-level matrix of tonal stimuli. In the matrix, the frequencies ranged from 4 to 44 kHz with 1 kHz increments, while the levels varied between 80 and 0 dB SPL with 10 dB decrements. The recording depth of population A1 neurons was within 400–700 μm below the pial surface, presumably in cortical layers 3 and 4 predominantly.

We initially determined the rate-level function of an A1 neuron under probe-only conditions (Figures 1B, H). The frequency of the probe was set at CF of the neuron whereas the levels of the probe were varied within the range of 0 to 80 dB SPL with 10 dB increments (Figure 1B). The detection threshold of the neuron was defined as the probe level that elicited a response equivalent to 20% of the maximum response in the rate-level function obtained under probe-only conditions (Figure 1H). Subsequently, we determined the rate-level functions of this A1 neuron under masker + probe conditions when masker and probe were presented at the same spatial azimuth (Figures 1C, H). The masker is a white noise, and the level of the masker was typically set to 10 dB above the detection threshold observed under probe alone conditions, ensuring a sufficient masking effect for investigating spatial release from masking when masker was moved away from the probe's azimuth. The masker level is a fixed level for each neuron, but can be varied across neurons due to different detection threshold under probe-only condition. In the present study, we focused on the analyzing the data of onset-responding neurons. The responses to the masker did not overlap with the responses to the probe. The neuronal responses to the probe were determined from the number of spikes evoked within the time window of the probe (i.e., 350 to 400 ms) under both probe-only condition and masker + probe conditions. Under masker + probe conditions, the detection threshold of the neuron was again defined as the probe levels corresponding to 20% of the maximum response in the rate-level functions obtained under probe-only conditions (referenced by the 20% line in Figure 1H). When the masker induced a detection threshold increase of at least 5 dB at the same azimuth as the probe, we fixed the probe azimuth and systematically varied the masker azimuth to determine the rate-level functions under these conditions (Figures 1D–H), thereby investigating spatial release from masking (Figures 1H–J).

### 2.4 *In vivo* whole-cell patch clamp recordings

*In vivo* whole-cell recordings were conducted with a MultiClamp 700B amplifier and a Digidata-1440A data acquisition system (Molecular Devices). The patch pipettes contained the following components (in mM): 125 Cs-gluconate, 10 HEPES, 0.5 EGTA, 2 CsCl, 5 TEA-Cl, 4 MgATP, 0.3 GTP, 10 phosphocreatine, 3.5 QX-314, with pH adjusted to 7.2 and osmolarity adjusted to 295 mOsm (Cai et al., 2018; Xie et al., 2018). The impedance of the patch pipettes was maintained at approximately 6–7 MΩ. The pipettes were advanced perpendicularly to the auditory cortex via MX-7600 manipulator (Siskiyou Corporation). Whole-cell and pipette capacitances were compensated, and the initial series resistance (20–50 MΩ) was compensated for 50% to achieve an effective series resistance of 10–25 MΩ. The signal sample rate was 10 kHz, and the data were low-pass filtered at 5 kHz and analyzed with Clampfit 10 (Molecular Devices). In the current-clamp mode, if the initial resting membrane potential of the patched neuron fell below −45 mV, the recording was switched to voltage-clamp mode to determine the sound evoked postsynaptic currents (PSCs). The patched neurons were voltage-clamped at 0 mV to measure the inhibitory postsynpatic currents (IPSCs), and −70 mV to measure the excitatory postsynaptic currents (EPSCs). The data of a cell were excluded if either the initial resting membrane potential was less than −45 mV or the series resistance changed more than 30% over the entire experiment (Xie et al., 2018). We define the CF of the neuron as the tonal stimulus frequency that could elicite EPSC at the lowest sound level.

The parameters for the probe and masker stimuli used in the whole-cell recording were similar to those employed in the extracellular recording. The spatial azimuth of the probe was fixed at C80, while the spatial azimuths of the masker were varied at C80, C40, 0, I40, and I80. The frequency of the probe was set at the CF of the patched neuron. Under probe-only and masker + probe conditions, we recorded the EPSC and IPSC elicited by the probe within the level ranges of 0 to 80 dB SPL, with increments of 10 dB. Each stimulus condition was repeated ten times and the PSC traces were averaged. The PSC threshold was defined as the tonal stimulus level corresponding to the 20% of the maximum peak amplitude of the PSC in the function of PSC peak amplitude vs. level under probe-only condition. The masker level was set to 10 dB above the EPSC threshold obtained under probe-only conditions. The recording depth of A1 neurons in whole-cell recording was within 400–700 μm below the pial surface.

### 2.5 Population data analysis

The population data analysis were performed using the Kruskal-Wallis test and Mann–Whitney test for non-parametric independent samples, as well as the Friedman test and Wilcoxon Signed Rank test for non-parametric related samples. It was considered as having significant differences between groups when *p* < 0.05.

## 3 Results

In the extracellular recording study, we examined the spatial release from masking in 201 neurons derived from 122 rats. The characteristic frequencies (CFs) of these neurons ranged from 6 to 39 kHz (mean ± SD: 22.3 ± 10.6 kHz). The minimum thresholds for these neurons varied between 0 and 46.4 dB SPL (mean ± SD: 22.8 ± 11.8 dB SPL). Due to the time-consuming nature of data collection and the restricted recording duration for each neuron, we encountered cases where data could not be collected for all three designed probe azimuths prior to the death of the recored neurons. Consequently, we assessed the effects of spatial release from masking under two probe azimuths conditions in 25 neurons, three probe azimuths conditions in 10 neurons, while the remaining 166 neurons were evaluated under only one probe azimuth condition.

### 3.1 The effect of spatial release from masking in a representative A1 neuron

In this study, we investigated the effect of spatial release from masking by analyzing the responses of A1 neurons to probe stimuli under different conditions. Specifically, we compared the conditions where the masker and probe were co-located at the same azimuth with those where they were spatially separated. Figure 1 illustrates the dot raster plots depicting the responses of a representative A1 neuron to probe stimuli under different masker conditions, highlighting the spatial release from masking effect. The probe stimuli were consistently positioned at azimuth C80, while the masker stimuli were varied across multiple azimuths: C80, C40, 0, I40, and I80. When both the masker and probe were located at C80, this neuron exhibited significantly reduced responses to probe stimuli at lower stimulus levels (below 50 dB SPL) under masker + probe conditions compared to probe-only conditions (Figures 1B, C). However, as the masker was displaced from the probe's azimuth (C80), the neuron's responses to probe stimuli recovered to levels comparable to those observed under probe-only conditions (Figures 1D–G). We determined the detection thresholds for this neuron using rate-level functions obtained under various acoustic stimulus conditions (Figure 1H). The detection threshold was defined as the stimulus level corresponding to 20% of the maximum response in the rate-level function derived from probe-only conditions (see the dotted reference line). For this particular neuron, the detection threshold was 12.7 dB SPL under probe-only condition. Under masker + probe conditions, when both masker and probe were positioned at azimuth C80, the detection threshold increased to 30.4 dB SPL, indicating a threshold shift of 17.7 dB relative to the probe-only condition. Conversely, when the probe remained fixed at azimuth C80 while the masker was moved to azimuths C40, 0, I40, and I80, the detection thresholds were 11.4 dB SPL at azimuth C40, 8.2 dB SPL at azimuth 0, 13.0 dB SPL at azimuth I40, and 13.9 dB SPL at azimuth I80 (Figures 1H, I). These results demonstrate the spatial release from masking when the azimuths of the probe and masker are not aligned. The maximum masking release was observed when the masker was positioned at azimuth 0. To assess the impact of spatial release from masking for population neurons, we quantified the threshold decrease for each A1 neuron across different masker azimuth conditions relative to the detection threshold when the masker and probe azimuths were aligned (Figure 1J). The threshold decrease was calculated as follows: the detection threshold determined under the condition where the masker and probe azimuths were aligned minus the detection threshold determined under conditions where the masker and probe were positioned at different azimuths. A positive value of threshold decrease indicates spatial release from masking. For this representative neuron, with the probe azimuth fixed at C80, moving the masker away from azimuth C80 to other spatial azimuths resulted in a threshold decrease of 19.0 dB at azimuth C40, 22.2 dB at azimuth 0, 17.4 dB at azimuth I40, and 16.5 dB at azimuth I80. These data unequivocally demonstrate the effect of spatial release from masking.

### 3.2 Population A1 neurons demonstrating spatial release from masking

**Figure 2:**
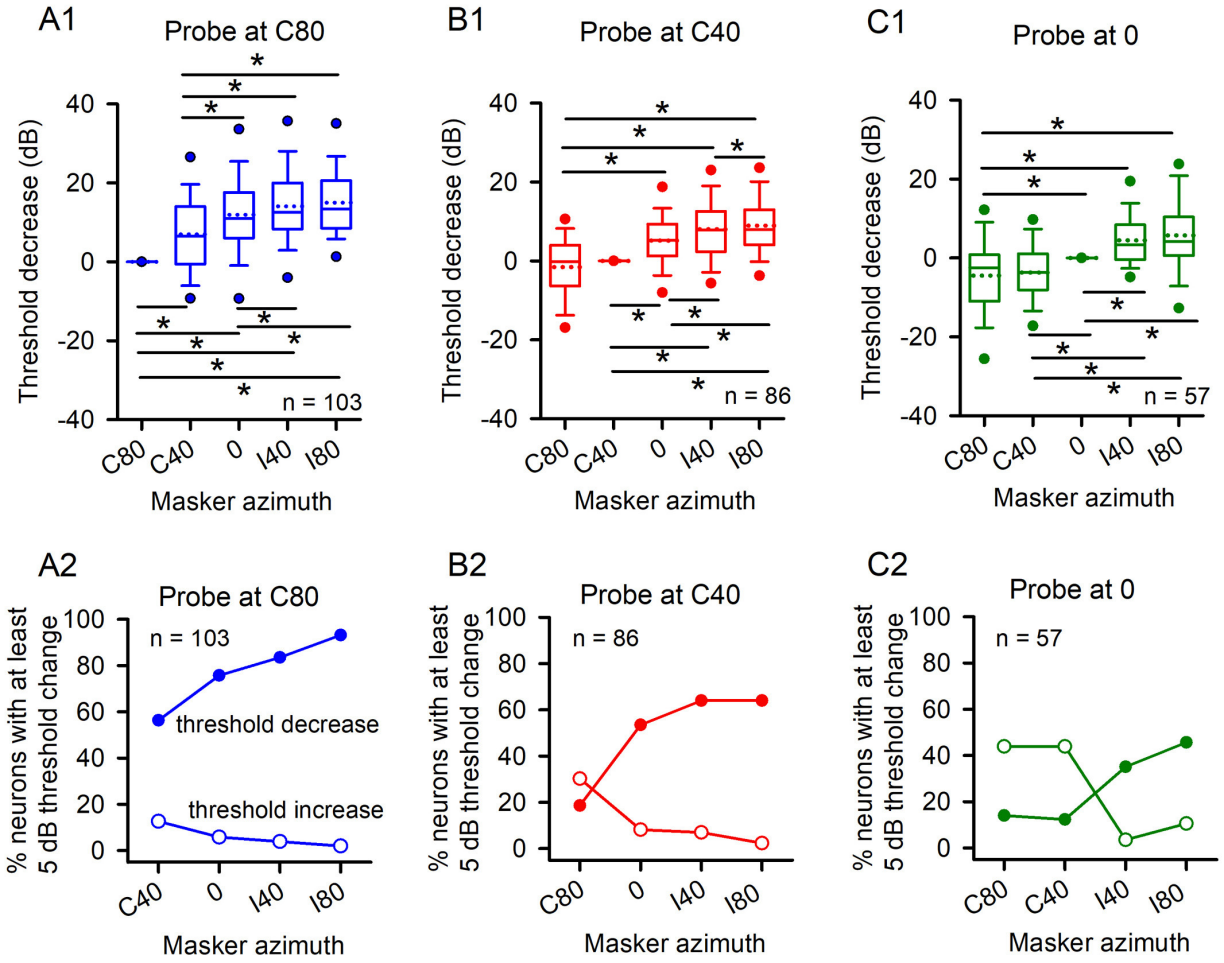
The population data of A1 neurons showing threshold changes with varying spatial azimuth of both the masker and the probe. The threshold decrease was normalized to zero when both the masker and the probe were aligned in azimuth. For the threshold decrease, positive values indicate masking release while negative values indicating no masking release. The spatial azimuth of the masker varied from C80 to I80. **(A1, A2)** The probe was fixed at C80; **(B1, B2)** The probe was fixed at C40; **(C1, C2)**, the probe was fixed at 0. **(A2, B2, C2)** Percentages of A1 neurons showing threshold decreases (filled circles) and threshold increases (unfilled circles) determined at various masker azimuths. The “*” indicates significant differences for data between groups. The “*n*” represents number of neurons.

Previous studies have shown that the majority of A1 neurons exhibit azimuth selectivity, and respond strongly to stimuli presented from contralateral azimuths but weakly to stimuli presented from ipsilateral azimuths (Phillips and Irvine, 1983; Kelly and Sally, 1988; Rutkowski et al., 2000; Ojima and Murakami, 2002; Higgins et al., 2010; Razak and Fuzessery, 2010; Yao et al., 2013; Gao et al., 2017, 2018; Wang et al., 2019). Therefore, in this study we investigated the spatial release from masking by presenting probe stimuli at azimuths C80, C40, and 0. Figure 2 illustrates the data from population A1 neurons. When the probes were positioned at azimuth C80 and the maskers were moved from azimuth C80 to C40, 0, I40 and I80, there was a significant decrease in detection thresholds among population A1 neurons (Figure 2A1, Wilcoxon Signed Ranks Test, C80 vs. C40, z = −5.746, *p* < 0.001; C80 vs. 0, z = −7.580, *p* < 0.001; C80 vs. I40, z = −8.307, *p* < 0.001; C80 vs. I80, z = −8.548, *p* < 0.001). These data indicate a substantial spatial release from masking. Further analysis revealed an increasing trend in the degree of threshold decrease as the masker was moved from the azimuth positions in the contralateral hemifield to ipsilateral hemifield, which demonstrats an overall increase in the spatial release from masking by expanding the spatial separation between masker and probe (Figure 2A1, Wilcoxon Signed Ranks Test, C40 vs. 0, z = −5.746, *p* < 0.001; C40 vs. I40, z = −7.104, *p* < 0.001; C40 vs. I80, z = −5.746, *p* < 0.001; 0 vs. I40, z = −3.09, *p* = 0.002, 0 vs. I80, z = −3.578, *p* < 0.001). When probe stimuli were presented at azimuth C40 and maskers were shifted from azimuth C40 to 0, I40, and I80, a marked decrease in detection thresholds was noted (Figure 2B1, Wilcoxon Signed Ranks Test, C40 vs. 0, z = −5.852, *p* < 0.001; C40 vs. I40, z = −6.764, *p* < 0.001; C40 vs. I80, z = −7.417, *p* < 0.001). Similarly, when probe stimuli were presented at frontal azimuth 0 and maskers were moved from azimuth 0 to I40 and I80, significant decreases in detection thresholds were also observed (Figure 2C1, Wilcoxon Signed Ranks Test, azimuth 0 vs. I40, z = −4.173, *p* < 0.001; azimuth 0 vs. I80, z = −4.040, *p* < 0.001). Furthermore, an increase in spatial release from masking was evident as the spatial separation between the masker and probe increased from the azimuth positions in the contralateral hemifield to the ipsilaterallateral hemifield (Figure 2B1, Wilcoxon Signed Ranks Test, I40 vs. 0, z = −3.434, *p* = 0.001; I80 vs. 0, z = −4.459, *p* < 0.001; I80 vs. I40, z = −1.956, *p* = 0.049). However, no significant differences in threshold decrease were observed when the probe was positioned at azimuth C40 and maskers were moved from azimuth C40 to C80 (Figure 2B1, Wilcoxon Signed Ranks Test, C80 vs. C40, z = −1.098, *p* = 0.272). Additionally, when probe stimuli were presented at frontal azimuth 0 and maskers were shifted from azimuth 0 to C40 and C80, a stronger masking effect was observed in population neurons when the maskers were located at azimuths C40 and C80 compared to when masker was positioned at azimuth 0 (Figure 2C1, Wilcoxon Signed Ranks Test, C80 vs. 0, z = −3.267, *p* = 0.001; C40 vs. 0, z = −3.353, *p* = 0.001; C80 vs. C40, z = −0.259, *p* = 0.795). These data indicate that the spatial release from masking effect was not evident when the maskers were moved from azimuth 0 to the azimuths in the contralateral hemifield.

To exam the influence of spatial separation between the masker and probe on the population of A1 neurons involved in spatial release from masking, we analyzed the proportions of A1 neurons that exhibited at least a 5 dB threshold decrease at each masker azimuth. These data indicate that the proportion of A1 neurons exhibiting spatial release from masking increases as the azimuthal separation between the probe and masker expands, particularly toward the masker azimuths in the ipsilateral hemifield (Figures 2A2, B2, C2, filled circles). Notably, when the probe was positioned at azimuth C80, more than half of the tested A1 neurons exhibited spatial release from masking even when the masker was situated at azimuths in the contralateral hemifield (Figure 2A2, azimuth C40). Additionally, we also analyzed the proprotion of A1 neurons that exhibited at least a 5 dB threshold increase at each masker azimuth (Figures 2A2, B2, C2, unfilled circles). These data indicated a higher proportion of A1 neurons showing the masking effect at azimuths in the contralalteral hemifield than in the ipsilateral hemifield. Overall, the data presented in Figure 2 indicate that the effect of spatial release from masking becomes increasingly pronounced as maskers are displaced from azimuth positions colocalized with the probe toward azimuth positions within the ipsilateral hemifield (Figure 2).

### 3.3 The spatial release from masking and the spatial azimuth preference of A1 neurons

**Figure 3:**
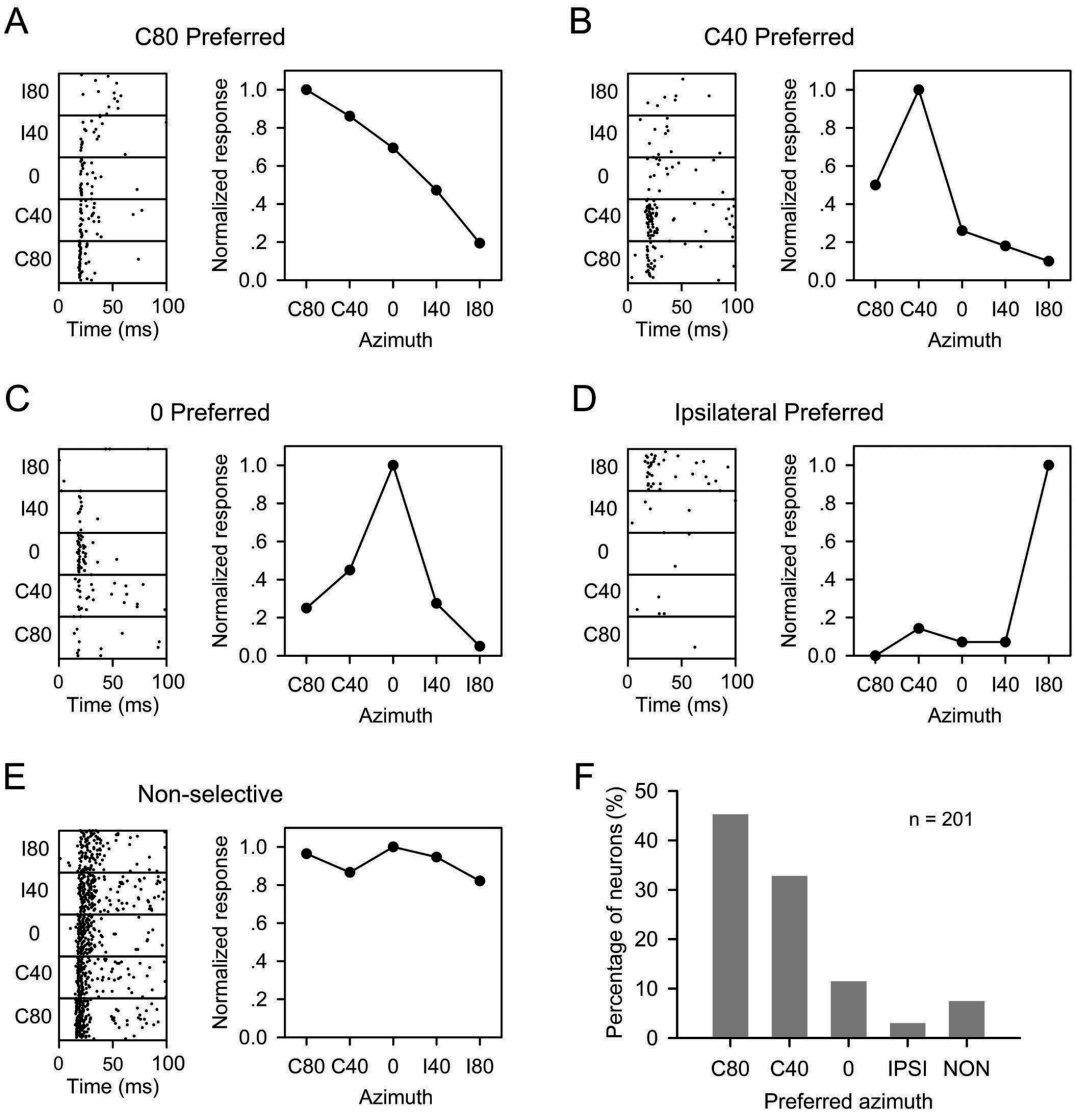
The azimuth preference of rat A1 neurons. The azimuths include C80, C40, 0, I40, and I80. **(A–E)** Dot raster plots and line plots for five representative A1 neurons responding to sound stimuli from different azimuths. The categories of the azimuth preference are as follows: C80-preferred **(A)**, C40-preferred **(B)**, 0-preferred **(C)**, ipsilateral preferred **(D)**, and non-selective **(E)**. **(F)** The percentage of neurons within each category of azimuth preferences. “*n*” indicates total number of neurons.

To investigate whether the effect of spatial release from masking is associated with the spatial azimuth preference of A1 neurons, we systematically recorded the responses of each A1 neuron to noise-only stimuli presented at five azimuths: C80, C40, 0, I40, and I80. The parameters of these noise stimuli were identical to those used for the masker stimuli in each A1 neuron. We classified the azimuth preference of A1 neurons into distinct categories based on these responses, and subsequently analyzed the effect of spatial release from masking for population A1 neurons within each category. Specifically, an A1 neuron was categorized as exhibiting azimuth preference if the difference between its maximum and minimum response across different azimuths exceeded 20% of the maximum response. Conversely, neurons that failed to meet this criterion were classified as non-selective to spatial azimuth. For example, an A1 neuron was categorized as C80-prefered if it exhibited the strongest response to stimuli presented from azimuth C80 and met the aforementioned criterion (Figure 3A). Similarly, A1 neurons were classified as C40-preferred (Figure 3B), 0-preferred (Figure 3C), and ipsilateral preferred (Figure 3D) based on their strongest responses at corresponding azimuths and compliance with the preference criterion. Neuron E in Figure 3 exhibited non-selective to spatial azimuth (Figure 3E), as the difference between the maximum and minimum responses in the response vs. azimuth function did not exceed 20% of the maximum response. Among the total of 201 A1 neurons tested, the majority (89.55%, 180/201) preferred azimuths in the contralateral hemifield (C80 and C40) and azimuth 0, while only a small proportion of neurons (2.99%, 6/201) exhibited preferences to azimuths in the ipsilateral hemifield, i.e., azimuth I40 and I80 (Figure 3F). Additionaly, less than 10% (7.46%, 15/201) of A1 neurons displayed non-selective to spatial azimuth (Figure 3F).

**Figure 4:**
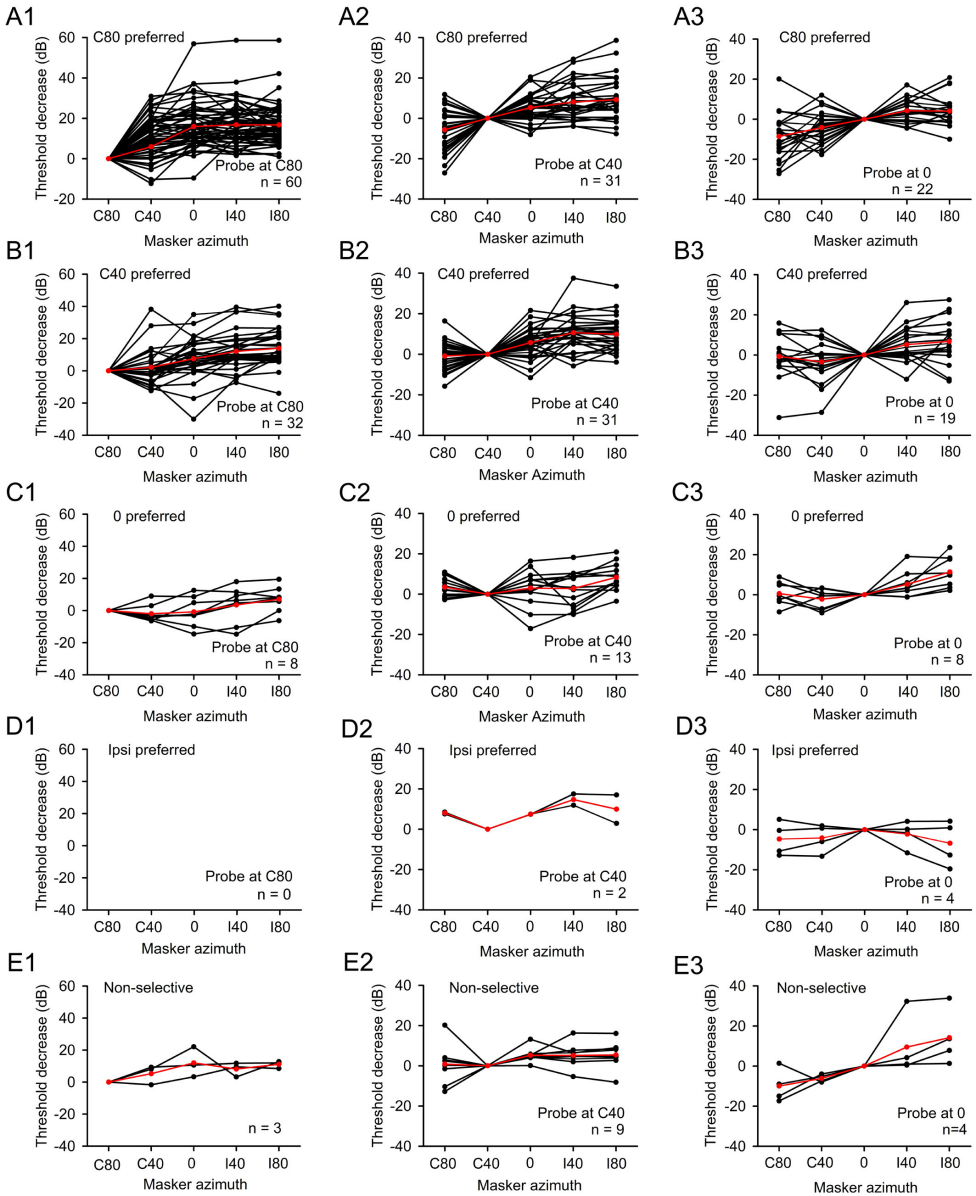
The spatial release from masking of A1 neurons with various spatial azimuth selectivity. Panels in each row show the data of A1 neurons in one category of azimuth selectivity. Panels in each column show the data of neurons when probes were positioned at one spatial azimuth. **(A1, B1, C1, D1, E1)** Probes were positioned at azimuth C80; **(A2, B2, C2, D2, E2)** Probes were positioned at azimuth C40 (middle column); **(A3, B3, C3, D3, E3)** Probes were positioned at azimuth 0. Each line drawing with black filled circles displays the data of one neuron. Red filled circles show the mean values for the population neurons determined under specific conditions.

For A1 neurons categorized within each azimuth preference group, we quantitatively analyzed the threshold decrease of each A1 neuron at each masker azimuth (Figure 4). Overall, the majority of A1 neurons exhibited spatial release from masking (i.e., a positive threshold decrease) when the maskers were displaced from the azimuths colocalized with the probe toward the azimuths in the ipsilateral hemifield. This phenomenon was observed for neurons with azimuth preferences at C80 (Figures 4A1–A3), C40 (Figures 4B1–B3), and 0 (Figures 4C1–C3). However, when the maskers were moved from the the azimuths where masker and probe were colocalized toward the azimuths in the contralateral hemifield, most A1 neurons did not exhibit significant spatial release from masking (Figures 4A2, A3 for C80 preferred neurons, Figures 4B2, B3 for C40 preferred neurons, and Figure 4C3 for 0 preferred neurons). For the A1 neurons exhibiting non-selective to spatial azimuth, the majority demonstrated spatial release from masking when the masker was moved from azimuth C80 to I80 at all tested probe azimuths (Figures 4E1–E3). Given the limited number of ipsilateral preferred A1 neurons collected (Figures 4D1–D3), further analysis for these neurons was not conducted. Generally, the mean threshold decrease for populations AI neurons increased when masker was moved from aligned azimuths with probes to azimuths in the ipsilateral hemifield (see the data in Figure 4 in each panel with red lines and filled circles). The data presented in Figure 4 indicate that the spatial release from masking of rat A1 neurons is not entirely associated with their azimuth preferrence. While the effect of spatial release from masking varies among A1 neurons, the spatial release from masking typically occurs when the masker is displaced away from the probe azimuths, particularly when moved from azimuths in the contralateral hemifield toward the azimuths in the ipsilateral hemifield, regardless of the azimuth preferrences of A1 neurons.

**Figure 5:**
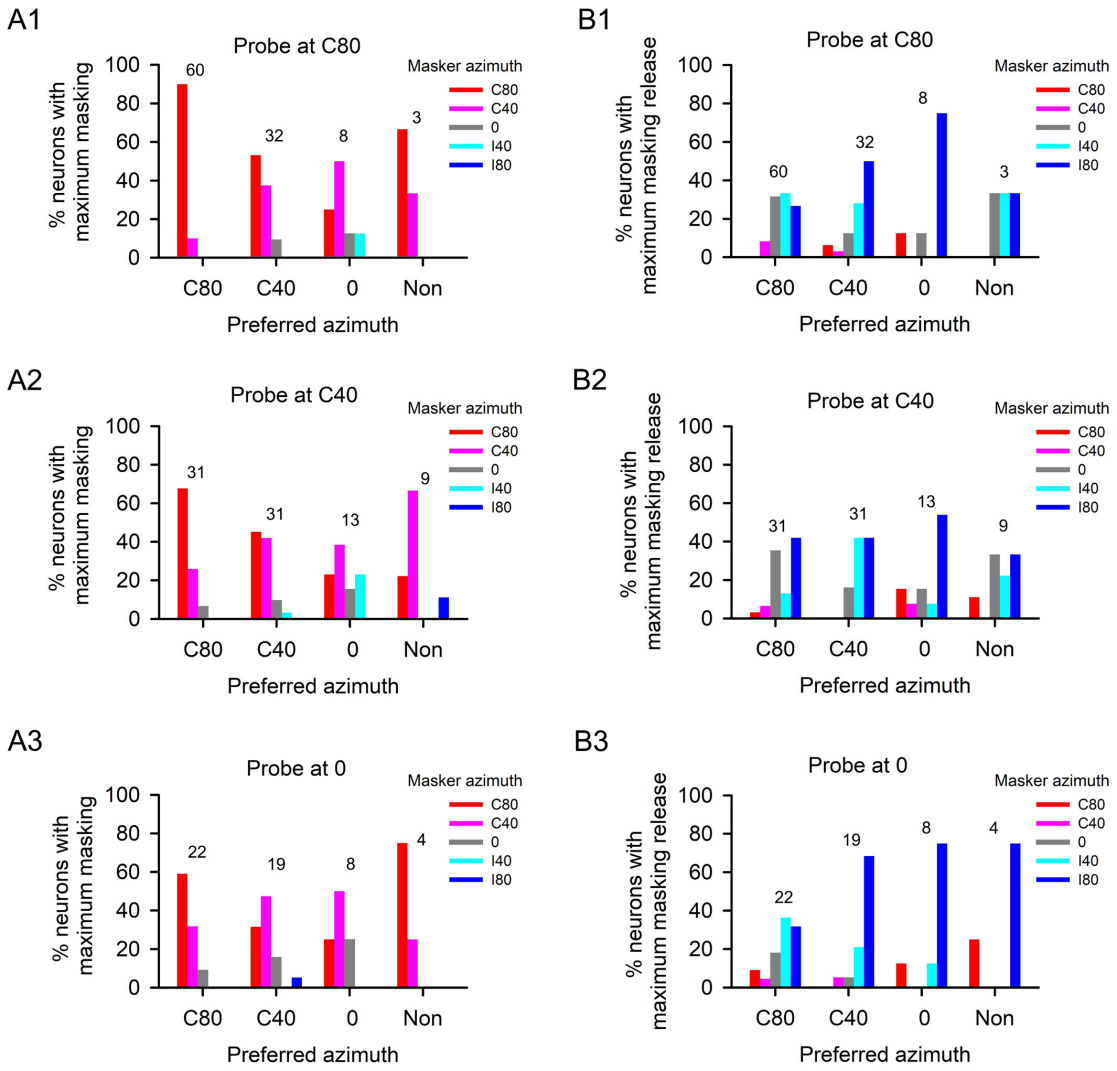
Percentages of neurons with maximum masking and maximum masking release when masker azimuths were varied. The abscissa shows the categories of neurons with different azimuth preference. **(A1, B1)** The probe was fixed at azimuth C80; **(A2, B2)** The probe was fixed at azimuth C40; **(A3, B3)** The probe was fixed at azimuth 0. Numbers shown on the top of the bars show the number of neurons at each category of preferred azimuth.

For each A1 neuron, we conducted an in-depth analysis to identify the masker azimuth that elicited the greatest masking effect (i.e., the largest threshold shift) comparing to probe-only condition) and the masker azimuth that induces the maximum masking release (i.e., the largest threshold decrease). Subsequently, we investigated the relationship between these masker azimuths and the preferred azimuths of the A1 neuron population. Given the limited number of neurons that preferred to ipsilateral azimuths (I80 and I40), our analysis focused on neurons with the following azimuth preferences: C80, C40, 0, and non-selective to spatial azimuth. In general, across these azimuth preferences of A1 neurons, the strongest masking effect was consistently observed when the maskers were positioned in the azimuths within the contralateral hemifield (C80 and C40), irrespective of the tested probe azimuths (Figures 5A1–A3). Additionally, when the probes were presented at azimuth C80, a minor proportion of C40-preferred and 0-preferred neurons exhibited the strongest masking effect when the maskers were located at azimuth 0 or I40 (Figure 5A1). Similarly, when the probes were presented at azimuths C40 and 0 (Figures 5A2, A3), a small fraction of neurons with preferences for azimuths C80, C40, and 0 showed the strongest masking effect when the maskers were situated at azimuth 0. In a few cases, the strongest masking effects were also observed when the maskers were presented at azimuths I40 or I80 (Figures 5A1–A3).

Regarding the spatial release from masking, irrespective of whether the probes were positioned at any of the three azimuths (i.e., C80, C40, or 0), the majority of A1 neurons consistently demonstrated the largest effect of spatial release from masking when the maskers were located at the azimuths in the ipsilateral hemifield (I40 and I80) (Figures 5B1–B3). Additionally, the maximum spatial release from masking was also observed in certain neurons when the maskers were located at other azimuths. Specifially, when the probes were positioned at azimuths C80 and C40, the greatest effect of spatial release from masking was evident for some A1 neurons when the maskers were presented at azimuth 0 (Figures 5B1, B2). When the probes were positioned at azimuth 0, the maximum spatial release from masking was observed for a few neurons (with preferred azimuths at C80 and 0, or non-selective to spatial azimuth) when the maskers were at azimuth C80, and for a few neurons (with preferred azimuths at C80 and C40) when the maskers were at azimuth C40 (Figure 5B3). The data in Figure 5 suggest that, for the majority of A1 neurons, regardless of their azimuth selectivity, the maximum spatial release from maskingt was consistently observed when the masker was shifted from the azimuths in the contralateral hemifield or azimuth 0 toward the azimuths in the ipsilateral hemifield, irrespective of the probe position at azimuths C80, C40, and 0.

### 3.4 The synaptic representation of spatial release from masking

**Figure 6:**
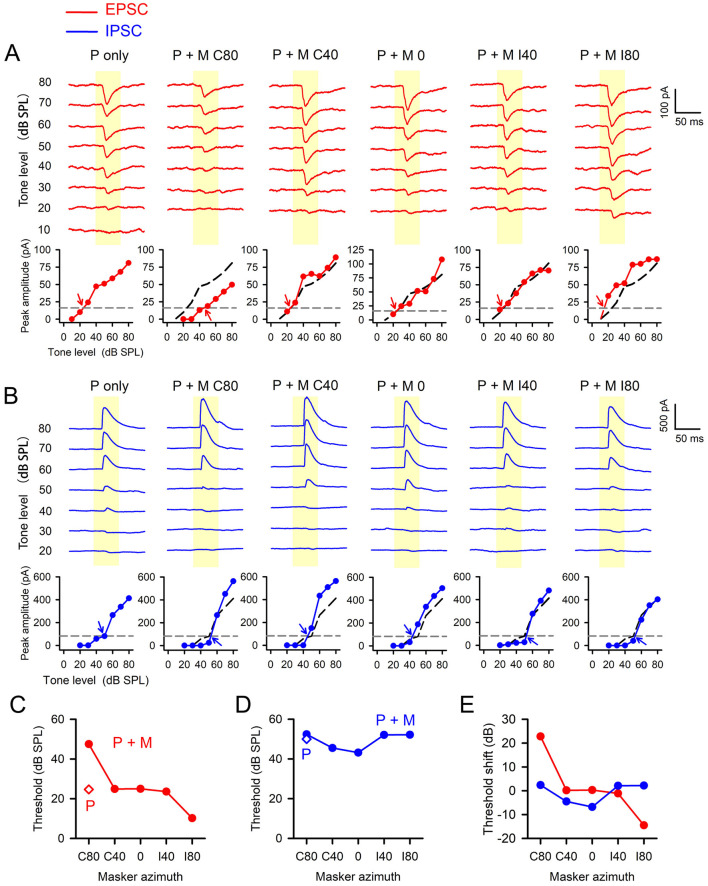
The data obtained from a representative A1 neuron show the variations in EPSCs and IPSCs determined under different stimulus conditions. P, probe only; P + M, probe + masker. Yellow columns show the duration of the probe. The spatial azimuth of the probe was fixed at C80, whereas the masker azimuths were set at C80, C40, 0, I40, and I80 respectively. **(A)** The upper portion displays traces of EPSCs determined at different conditions, while the lower portion illustrates the functions of EPSC peak amplitude vs. sound level. The gray dash lines represent the reference lines corresponding to 20% of maximum EPSC amplitude at probe-only condition. At each stimulus condition, the EPSC threshold was defined as the sound level at which this reference line intersects with the corresponding EPSC peak amplitue vs. level function (see arrowhead). The dark dash curve is identical to the curve showing at probe-only condition for comparison. **(B)** The illustrations for the traces of IPSCs and the IPSC peak amplitude vs. sound level functions determined under various stimulus conditions. The method for determining the IPSC threshold is similar to that determining the EPSC threshold. **(C, D)** The EPSC thresholds **(C)** and IPSC thresholds **(D)** determined under different stimulus conditions. **(E)** The normalized EPSC threshold shifts and IPSC threshold shifts measured relative to their corresponding thresholds obtained under probe-only condition.

The results from the extracellular recordings indicate that the masking effect on the responses of A1 neurons to probes is influenced by the spatial azimuth of the masker. Specifically, when the probes were located at the azimuths in the contralateral hemifield or azimuth 0, a spatial release from masking was evident as the maskers shifted from azimuths in the contralateral hemifield to the azimuths in the ipsilateral hemifield. What, then, are the synaptic mechanisms responsible for this spatial release from masking? To address this issue, we used *in vivo* whole-cell patch clamp recording method to measure the EPSC and IPSC of A1 neurons to probes under both probe-only conditions and masker + probe conditions, with variations in masker azimuth. The data presented in Figure 6 depict the postsynaptic currents of an A1 neuron in response to probes under these conditions. The probes were positioned at azimuth C80 while the maskers were shifted from azimuth C80 to I80. The EPSC curves varied with both probe levels and masker azimuth (Figure 6A). The peak amplitue of EPSC decreased as probe level decreased. The EPSC threshold was defined as the sound level corresponding to the 20% of the maximum EPSC amplitude in the EPSC peak amplitude vs. tone level function. Consequently, the EPSC threshold determined under probe-only condition was 24.8 dB SPL (Figure 6A, probe-only, Figure 6C). When the masker and probe were presented at the same azimuth (C80), the EPSC amplitude vs. tone level curve shifted to the right relative to the probe-only condition, and the EPSC threshold increased to 47.6 dB SPL (Figure 6A, P + M C80, Figure 6C). When the masker was shifted from azimuth C80 to C40, 0, I40, and I80, the EPSC thresholds decreased to 24.9 dB SPL (Figure 6A, P + M C40, Figure 6C), 25 dB SPL (Figure 6A, P + M 0, Figure 6C), 23.6 dB SPL (Figure 6A, P + M I40, Figure 6C) and 10.2 dB SPL (Figure 6A, P+M I80, Figure 6C) repectively. Therefore, when the masker azimuth was moved away from the probe's azimuth to other spatial azimuths, the EPSCs exhibited a decrease in EPSC threshold, demonstrating the spatial release from masking in EPSC. For the IPSC, the IPSC curves and the peak amplitude of IPSC vs. probe level functions of this neuron in response to probe stimuli varied with the the probe level as well as the azimuth locations of the masker (Figure 6B). The IPSC threshold was defined as the sound level corresponding to the 20% of the maximum IPSC amplitude in the IPSC peak amplitude vs. tone level function. Under probe-only condition, the IPSC threshold of this neuron was higher than the EPSC threshold, reaching to 50 dB SPL (Figure 6B, P only, Figure 6D). When the probe and masker were both located at azimuth C80, the IPSC threshold slightly increased to 52.4 dB SPL (Figure 6B, P + M C80, Figure 6D). As the masker was move away from azimuth C80 toward I80, the IPSC threshold changed to 45.5 dB SPL at masker azimuth C40 (Figure 6B, P + M C40, Figure 6D), 43.2 dB SPL at masker azimuth 0 (Figure 6B, P + M 0, Figure 6D), 52.1 dB SPL at masker azimuth I40 (Figure 6B, P+ M I40, Figure 6D) and 52.2 dB SPL at masker azimuth I80 (Figure 6B, P+ M I80, Figure 6D), respectively.

Given that the EPSC threshold and IPSC threshold vary among neurons, to investigate the spatial release from masking at the synaptic level for a population of neurons, for each A1 neuron, we calculated the EPSC threshold shift under masker + probe conditions by subtracting the EPSC threshold under probe-only conditions from the EPSC threshold under masker + probe conditions. Similarly, the IPSC threshold shifts under masker + probe conditions were determined by subtracting the IPSC threshold under probe-only condition from the IPSC threshold under masker + probe conditions (Figure 6E). For the example neuron depicted in Figure 6, when both the masker and the probe were aligned at azimuth C80, the EPSC threshold shift was 22.8 dB. When the masker was displaced from azimuth C80 to C40, 0, I40, and I80, the EPSC threshold shift decreased to 0.1 dB at azimuth C40, 0.2 dB at azimuth 0, −1.2 dB at azimuth I40, and −14.6 dB SPL at azimuth I80 (Figure 6E). For the IPSC threshold shift, when the probe and the masker were both at azimuth C80, the IPSC threshold shift was 2.4 dB at azimuth C80. When the masker was displaced from azimuth C80 to C40, 0, I40, and I80, the IPSC threshold shift decreased to −4.5 dB at azimuth C40, −6.8 dB at azimuth 0, 0.1 dB at azimuth I40, and 0.2 dB at azimuth I80 (Figure 6E). These data indicate that when the maskers were displaced from the probe azimuth (C80) toward the azimuths in the ipsilateral hemifield, the EPSC shift of this neuron was more pronounced than the IPSC threshold shift.

**Figure 7:**
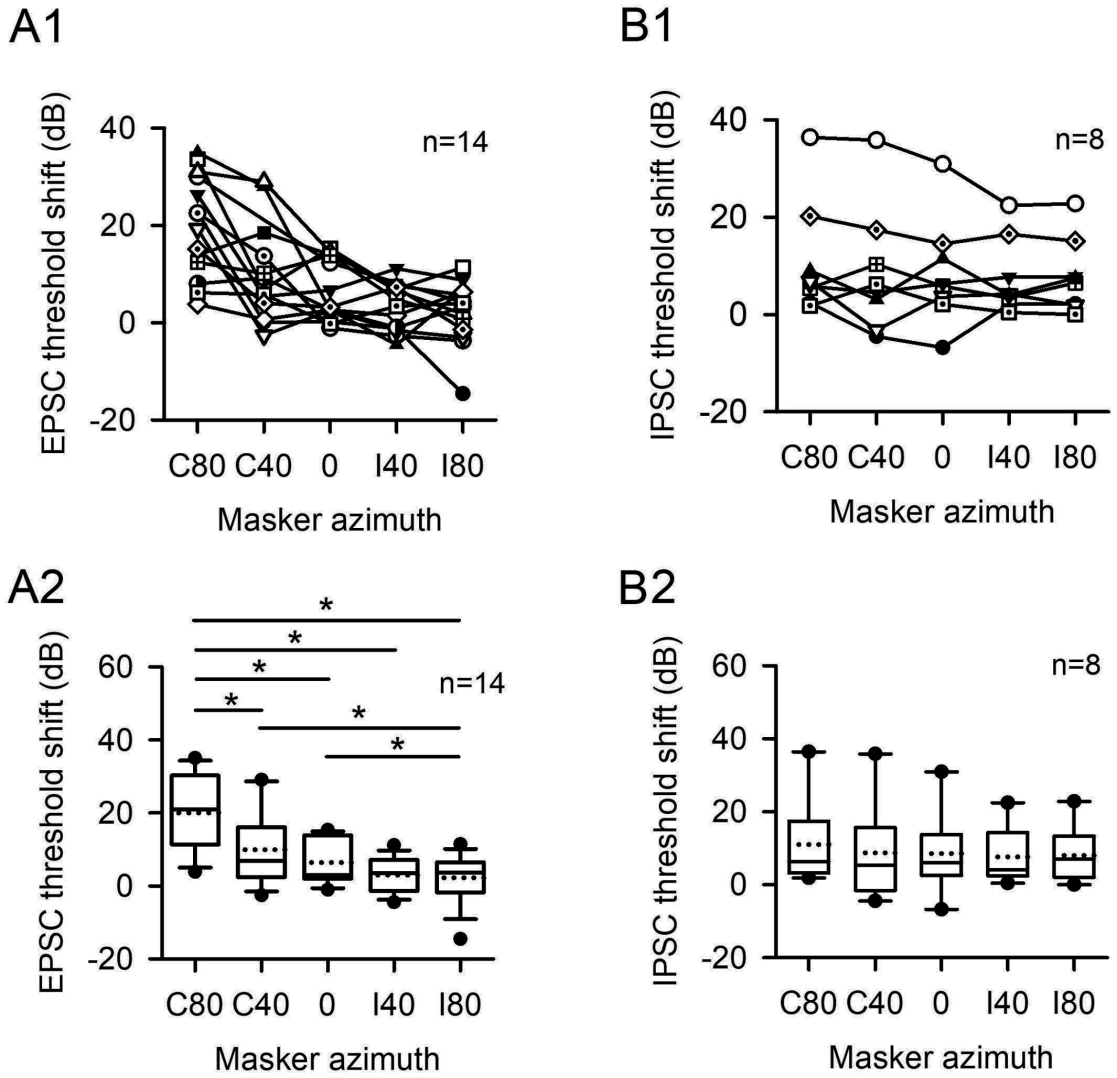
The EPSC threshold shift and IPSC threshold shift vary as a function of masker azimuth for population neurons. The probe azimuth was at C80. The PSC threshold shift was determined by subtracting the PSC threshold determined under probe-only conditions from the PSC threshold determined under probe + masker condition. **(A1, A2)** EPSC threshold shift; **(B1, B2)**, IPSC threshold shift. The “*n*” indicates the total number of neurons. The “*” indicates significant differences between two conditions (Wilcoxon Signed Ranks Test, *p* < 0.05).

We have quantified the EPSC threshold shift as a function of masker azimuth for 14 A1 neurons (Figures 7A1, A2). The EPSC threshold shift in A1 neurons tends to decrease as the masker moves from azimuth C80 (where probes are located) toward I80 (Friedman Test, *df* = 4, *x*^2^ = 21.544, *p* < 0.001). When the masker was positioned at azimuth C80, the EPSC threshold shifts were significantly larger compared to the other four azimuth positions (Wilcoxon Signed Ranks Test: C80 vs. C40, z = −2.621, *p* = 0.009; C80 vs. 0, z = −3.045, *p* = 0.002; C80 vs. I40, z = −3.180, *p* = 0.001; C80 vs. I80, z = −3.170, *p* = 0.002). Additionally, the threshold shift was generally smallest at azimuth I80 (Wilcoxon Signed Ranks Test: C40 vs. I80, z = −2.271, *p* = 0.023; 0 vs. I80, z = −2.040, *p* = 0.041), with the exception of the comparison between azimuth I40 and I80 (z = −0.559, *p* = 0.576). These findings indicate that the masking effect on EPSC is most pronounced when the probe and masker are aligned in azimuth, and spatial release from masking becomes evident as the masker is displaced from the probe.

We have determined the IPSC threshold shift for 8 of the 14 neurons (Figures 7B1, B2). Two neurons exhibited a significantly greater IPSC shift at azimuths in the contralateral hemifield and a smaller shift at azimuths in the ipsilateral hemifield, indicating a spatial release from masking in the IPSC (Figure 7B1, data with unfilled circles, or diamonds with dot). However, for the remaining six neurons, no systematic changes in IPSC threshold shift were observed as the masker was moved from azimuths in the contralateral hemifield toward the azimuths in the ipsilateral hemifield (Figure 7B1). For this population of eight neurons, the Friedman Test did not reveal statistically significant differences in the IPSC threshold shift across the five masker azimuth conditions (*df* = 4, *x*^2^ = 3.400, *p* = 0.493).

## 4 Discussion

Human and animals are adept at perceiving communication sounds in noisy and complex acoustic environments. The phenomenon of spatial release from masking occurs when the detection or discrimination of a probe is enhanced due to spatial separation between the probe and the masker (Saberi et al., 1991; Freyman et al., 1999; Hawley et al., 1999; Marrone et al., 2008). This effect can be observed in various auditory scenarios and plays a crucial role in our ability to perceive sounds in complex backgrounds. In this study, we utilized single-unit recording and *in vivo* whole-cell patch-clamp recording techniques to examine the impact of maskers from different spatial locations on the responses of A1 neurons to probes. We characterized the neuronal and synaptic representation of spatial release from masking by analyzing spike counts and postsynaptic current data of A1 neurons in response to probes presented with maskers at various azimuths. At the neuronal level, we found that the detection thresholds of most A1 neurons, as determined by spike counts in response to probes, were significantly decreased when maskers were displaced from azimuths colocalized with the probes to other separated azimuths in the ipsilateral hemifield, demonstrating the effect of spatial release from masking. Similarly, at the synaptic level, the detection thresholds of A1 neurons, as determined by the amplitude of evoked EPSCs in responses to probes presented at azimuths in the contralateral hemifield, were also decreased when maskers were shifted from azimuth positions in the contralateral hemifield to the ipsilateral hemifield relative to the recording site. Moreover, spatial release from masking was also observed for certain neurons when the masker was positioned at azimuths in the contralateral hemifield. Notably, the proportion of neurons showing the masking effect was higher when the masker was located in the contralateral hemifield than in the ipsilateral hemifield. To our knowledge, this is a first study providing both neuronal and synaptic evidences for spatial release from masking, which will enhance our understanding of its underlying mechanisms.

### 4.1 Spatial release from masking at neuronal level

Psychophysical studies have demonstrated that the detection threshold for a probe is higher when the probe and masker are colocalized compared to those when they are spatially separated (Misurelli and Litovsky, 2015; Srinivasan et al., 2016). In the current study, we observed that as the masker azimuth shifted from contralateral hemifield to ipsilateral hemifield, the detection thresholds for most A1 neurons in response to the probe gradually decreased, eventually reaching levels comparable to those under probe-only conditions. This finding demonstrates spatial release from masking at the cortical neuronal level, and aligns with previous psychophysical observations. However, a difference between the results from psychophysical studies and the results from this study is that, most A1 neurons did not exhibit pronounced spatial release from masking when the probe and masker were spatially separated in the contralateral auditory space. This observation may be attributed to the fact that most A1 neurons prefer stimuli from contralateral azimuths, resulting in a strong masking effect at these locations.

We further analyzed the relationship between the effect of spatial release from masking and the azimuth preference of A1 neurons. In the present study, most A1 neuron preferred stimuli from contralateral azimuths relative to the recording site, and only a small proportion of A1 neurons preferred stimuli from azimuths in the ipsilateral hemifield. This result is consistent with the previous findings in the auditory cortex of rats (Yao et al., 2013; Gao et al., 2018; Wang et al., 2019), cats (Furukawa and Middlebrooks, 2001), and primates (Ahissar et al., 1992). For the majority of rat A1 neurons determined in the present study, the most significant masking effect was observed when maskers were presented in the contralateral azimuths (C80 and C40), irrespective of the neurons' azimuth preference. This pattern also held true even for neurons that preferred stimuli from azimuth 0 or were non-selective to spatial azimuth. Conversely, most A1 neurons demonstrated the greatest spatial release from masking when masker was positioned at the azimuth in the ipsilateral hemifield (I40 and I80), regardless of their azimuth preference. As a result, when the masker is presented in the left auditory field, it exerts a more pronounced masking effect on the detection threshold of the probe for neurons in the right auditory cortex, while having a relatively weaker effect on those in the left auditory cortex. Conversely, when the masker is presented in the right auditory field, it has a stronger masking effect on the detection threshold of the probe for neurons in the left auditory cortex and a lesser impact on those in the right auditory cortex. This ensures that, regardless of the masker's location, one of the two hemispheres of the auditory cortex can effectively encode the probe, thereby enhancing the detection and perception of probe in noisy environments.

Previous animal studies have determined the neuronal correlates of spatial release from masking in the inferior colliculus of frogs (Ratnam and Feng, 1998; Lin and Feng, 2001) and cats (Lane and Delgutte, 2005), as well as in the auditory cortex of mice (Nocon et al., 2023), cats (Furukawa and Middlebrooks, 2001) and zebra finch (Maddox et al., 2012). The study of single-units in the inferior colliculus of cats revealed that the masked thresholds for population neurons sensitive to interaural time differences improved with the spatial separation of signal and masker, suggesting a neural basis for spatial release from masking in low-frequency sounds (Lane and Delgutte, 2005). Studies in the central auditory system of humans have observed spatial release from masking by recordings of auditory brainstem responses, frequency-following responses, and cortical auditory evoked potentials, demonstrating that the human brainstem and auditory cortex contribute to spatial release from masking (Rouhbakhsh et al., 2019; Li et al., 2024). In current study, we observed spatial release from masking effects in the auditory cortex, and it is plausible that some of these effects may originate from subcortical or intracortical processing stages.

### 4.2 Spatial release from masking at synaptic level

In this study, we examined spatial release from masking at synaptic level by analyzing the EPSC and IPSC threshold shifts in A1 neurons in response to probe while varying the azimuth of the masker. This is a first study of spatial release from masking at synaptic level in the auditory system. The EPSC threshold shift was the most pronounced when the masker and probe were colocalized in azimuth, decreasing as the masker were moved from azimuths in the contralateral hemifield to azimuths in the ipsilateral hemifield. These results indicate that subcortical auditory structures contribute to the spatial release from masking. Similar effects on the IPSC threshold shift were observed in only two neurons, indicating that the changes in inhibitory inputs with varying masker azimuth contribute to spatial release from masking of spike response in these specific neurons. In contrast, for other recorded neurons, the IPSC threshold shifts did not exhibit similar alterations with changes of masker azimuths. One potential explanation for this discrepancy is that variations in inhibitory influences from subcortical or intracortical neurons with spatial azimuth contribute to the observed spatial release from masking as the masker shifts from azimuths in contralateral hemifield toward ipsilateral hemifield.

### 4.3 Spatial release from masking and binaural hearing

Binaural hearing plays an important role in spatial release from masking. Effective spatial release from masking relies on accurate binaural cues, i.e. interaural time differences and interaural level differences. These binaural cues are essential for spatial separation and are processed by the auditory system to enhance speech intelligibility in noisy environments (Kidd et al., 2010; Ihlefeld and Litovsky, 2012), while conflicting cues reduce its effectiveness (Kidd et al., 2010; Ellinger et al., 2017). Degradation of the binaural cues, such as by covering one ear, significantly degrades spatial release from masking (Marrone et al., 2008). In addition, cochlear implant users exhibit reduced or absent spatial release from masking compared to normal-hearing listeners (Misurelli and Litovsky, 2012). This may be due to the inability to effectively utilize spatial cues, particularly interaural time differences (Ihlefeld and Litovsky, 2012). In the present study, the frequency component of the acoustic stimuli were at or above 4 kHz, so the interaural level difference is the major binaural cue contributing to the spatial release from masking observed at both neuronal and synaptic level in the auditory cortex.

### 4.4 Limitations in this study

We should acknowledge that in real-life noisy environments, such as cocktail parties, multiple maskers may originate from diverse spatial locations. As a result, this scenario is considerably more complex than the experiment design employed in the present study. Future research should aim to develop experiments to investigate the effects of spatial release from masking when multiple maskers are present in both the left and right auditory fields.

In the present study, we only used wideband noise as maskers. Previous studies have shown that both the types of maskers and the probe frequencies significantly influence the spatial release from masking, which in turn affects the ability to detect target sound in noisy environments (Johnstone and Litovsky, 2006; Klinge et al., 2011). Changes in harmonicity cues within maskers can also impact spatial release from masking (Klinge et al., 2011). In multi-talker listening scenarios, variations in vocal-characteristics influence the spatial release from masking (Oh et al., 2022, 2025). Under speech-speech masking conditions, differences in fundamental frequency can facilitate spatial separation in improving intelligibility (Yao et al., 2024). Moreover, greater spectral overlap between the masker and the probe has been associated with a more pronounced spatial release from masking (Best et al., 2013). Consequentially, the narrowband and broadband noise masker may exert different effects on spatial release from masking. Future studies should be conducted under diverse masking conditions to deepen our understanding of the neuronal and synaptic mechanism underlying spatial release from masking.

Another limitation of this study is that, due to technical challenges in precisely aligning the positions of the two speakers at identical azimuths and elevations, the elevation angle for the speaker presenting the probes was set at 0°, while that of the maskers was fixed at 10° across all tested conditions. This resulted in a 10° difference in elevation between the two speakers when their azimuths were aligned. However, given that the elevation of the maskers was consistently maintained at 10° throughout all tested conditions, the potential confounding effects of this experimental design on the conclusions of this study are expected to be minimal.

In the present study, the masker (400 ms duration) partially overlaps with the probe (50 ms duration), and the onset interval between the masker and probe is 300 ms. This stimulus paradigm simulates the scenario where the probe is presented against a background noise. Given this temporal relationship between masker and probe, the masker induces a forward masking effect on the response to the probe. However, the variation in the masker-evoked response may result in different degrees of masking effect on the response to the probe. Based on our current data, we cannot exclude the potential contributions of this factor when interpreting the observed effect of spatial release from masking. A previous study has demonstrated that forward masking in the auditory cortex is spatial broad, and changes in masker-evoked responses are not significantly correlated with alterations of suppressive effect on probe responses (Zhou and Wang, 2014). Furthermore, in this study, A1 neurons that are non-selective to spatial azimuth also exhibit spatial release from masking. Consequently, the spatial release from masking observed in this study is not entirely attributable to the azimuth preference of A1 neurons.

## 5 Conclusion

In summary, the findings of this study provide important evidence for spatial release from masking in the rat auditory cortex at both neuronal and synaptic levels. Compared to the detection thresholds of cortical neurons for probes under probe-only conditions as determined by spike counts and EPSCs, the detection thresholds significantly increased when the masker was co-localized with the probe. As the maskers were displaced from the azimuth of the probe toward azimuths in the ipsilateral hemifield relative to the recording site, the detection thresholds gradually decreased and recovered, demonstrating a spatial release from masking effect. These results will enhance our understanding of the neuronal and synaptic mechanisms underlying cortical processing of auditory signals in noisy environments.

## Data Availability

The original contributions presented in the study are included in the article/supplementary material, further inquiries can be directed to the corresponding author.
